# A Case of Concussion of the Brain Followed by Mental Derangement and Paralysis, Successfully Treated by Sulphuric Ether

**Published:** 1847-01

**Authors:** John Travis

**Affiliations:** Melville, Tennessee


Art. VI.—*A Case of Concussion of the Brain followed by Mental Derangement and Paralysis, successfully treated by Sulphuric Ether.* By John Travis, M. D., of Melville, Tennessee.

M. P. æt 25 years, was thrown from a horse against a dwelling house with great force; he was apparently dead for half an hour. His head was bruised and the integuments lacerat.

ed. When he revived he was found to be deranged in mind, in which state he continued for six weeks, notwithstanding he had two medical attendants with him daily. On examination, it was ascertained that there was no fracture of the cranium. So soon as his reason returned, his arms to the elbow, and his legs to the knees, became paralysed; he could move neither, which proved that the nerves of motion had lost their power. The function of the nerves of sensation remained unimpaired. In this condition his health otherwise was good. His physicians gave him a variety of medicines, and applied various rubefacients, blisters, &c., for several weeks, but the patient remained in *statu quo*. I was now called upon; I prescribed different articles of the materia medica—all in common use, in fact, except strychnia. This I intended to use as a dernier resort; but learning that ardent spirits would in a slight de gree excite the nerves of motion, I sent the patient sulphuric ether, and directed him to take a large tea-spoonful in cold water, three times a day. After using this medicine three days he could bear his weight on his feet; and in two weeks he could walk without any assistance, and is now in a manner well, having the use of his arms and legs.

I have thought it proper to make these facts known, as it is a mode of treatment that I believe has not hitherto been pursued, and one which has proved eminently successful in a case which was deemed hopeless by the patient and his friends.

November 12th, 1846.

